# The association between DNA methylation and exon expression in the Pacific oyster *Crassostrea gigas*

**DOI:** 10.1371/journal.pone.0185224

**Published:** 2017-09-25

**Authors:** Kai Song, Li Li, Guofan Zhang

**Affiliations:** 1 Key Laboratory of Experimental Marine Biology, Institute of Oceanology, Chinese Academy of Sciences, Qingdao, Shandong, China; 2 National & Local Joint Engineering Laboratory of Ecological Mariculture, Qingdao, Shandong, China; 3 Laboratory for Marine Fisheries and Aquaculture, Qingdao National Laboratory for Marine Science and Technology, Qingdao, Shandong, China; 4 Laboratory for Marine Biology and Biotechnology, Qingdao National Laboratory for Marine Science and Technology, Qingdao, Shandong, China; Xiamen University, CHINA

## Abstract

**Background:**

DNA methylation is one of the most important epigenetic modifications of eukaryotic genomes and is believed to play integral roles in diverse biological processes. Although DNA methylation has been well studied in mammals, data are limited in invertebrates, particularly Mollusca. The Pacific oyster *Crassostrea gigas* is an emerging genetic model for functional analysis of DNA methylation in Mollusca. Recent studies have shown that there is a positive association between methylation status and gene expression in *C*. *gigas*; however, whether this association exists at the exon level remains to be determined.

**Results:**

In this study, we characterized the genome-wide methylation pattern across two different tissues of *C*. *gigas* and found that methylated genes are expressed in more tissues and development stages than unmethylated genes. Furthermore, we found that different types of exons had different methylation levels, with the lowest methylation levels in the first exons, followed by the last exons, and the internal exons. We found that the exons included in the gene transcript contained significantly higher DNA methylation levels than skipped exons. We observed that the DNA methylation levels increased slowly after the start sites and end sites of exons seperately, and then decreased quickly towards the middle sites of exons. We also found that methylated exons were significantly longer than unmethylated exons.

**Conclusion:**

This study constitutes the first genome-wide analysis to show an association between exon-level DNA methylation and mRNA expression in the oyster. Our findings suggest that exon-level DNA methylation may play a role in the construction of alternative splicing by positively influencing exon inclusion during transcription.

## Background

DNA methylation is one of the most important epigenetic modifications of eukaryotic genomes and is believed to play integral roles in diverse biological processes, such as the regulation of temporal and spatial gene expression [1], alternative splicing [2], transcriptional noise control [3], and genome stabilization [4]. In most organisms studied, DNA methylation occurs predominantly at cytosine nucleotides, followed by guanine nucleotides or “CpG dinucleotides.” Despite the conserved units of DNA methylation, genome-wide methylated cytosine (mC) ratios are highly variable across animal taxa. The mC ratios of mammals are approximately one-order of magnitude greater than those observed in insects, such as silkworms, honeybees, and ants. For example, approximately 4–6% of genomic cytosine residues are methylated in humans [5, 6], whereas only 0.1–0.2% are methylated in insects [7–9]. Although the mC ratios are lower in invertebrates, DNA methylation is widespread and plays integral roles in their biological processes, i.e. gene expression regulation, development and reproduction [7, 9–16]. A recent study found that hyper- and hypomethylated genes were enriched, with functions linked to different biological processes [11, 17]. Hypermethylated genes are significantly enriched for biological processes related to metabolism and ubiquitous housekeeping functions of gene expression and translation. In contrast, hypomethylated genes associated with various developmental processes, cellular communication, and adhesion. DNA methylation is also implicated in the determination of gene expression levels and conservation status [18, 19]. Characterizing DNA methylomes in other poorly sampled taxa is essential to develop a better understanding of the evolution of DNA methylation as well as its functions and biological significance in eukaryotes.

MethylC-Seq technology couples the bisulfite-based detection of methylated cytosine residues with the use of high-throughput whole-genome sequencing. This technology was first applied to *Arabidopsis* [20], and then to more than 20 eukaryotic organisms, including fungi, plants, invertebrates, and vertebrates [5, 7, 13, 14, 21]. Application of this technology has enabled the identification of DNA methylation at single bases as well as new elaborate patterns and functional effects of DNA methylation. Although DNA methylation has been well studied in mammals, data are limited for invertebrates, particularly Mollusca. The diploid oyster *Crassostrea gigas* is used as a model organism for studies on immunology [22–25] and stress-response mechanisms [26–28]. The genome of *C*. *gigas* was published in 2012; this provided a basic resource for genetic and evolutionary studies in this species [29].

Analysis of CpG_O/E_ ratios have shown that DNA methylation is a common feature in the *C*. *gigas* genome, and some specific functional categories of genes have been shown to have significantly different levels of methylation, such as those involved in cell adhesion, cell-to-cell signaling, and signal transduction [12]. Furthermore, DNA methylation is known to play a functional role in this species; its absence in genes involved in the response to fluctuating conditions can facilitated phenotypic variation, which could contribute to increased adaptive potential [30]. High-throughput bisulfite sequencing has been used to better understand the role of DNA methylation in oysters on genome-wide and single-base nucleotide levels [31–33]. Those studies provided a comprehensive picture of the relationships between DNA methylation, CpG dinucleotides, genomic features, and gene expression. Recent studies in *Apis mellifera* (honeybee) and *Nasonia vitripennis* (jewel wasp) revealed a positive relationship between exon-level DNA methylation and mRNA expression, demonstrating that exon-specific DNA methylation may be associated with alternative splicing events [7, 16, 34]. Thus far, high-throughput bisulfite sequencing has been used to determine the characteristic DNA methylation at the gene level for *C*. *gigas*; however, studies comparing DNA methylation patterns at the exon level and those investigating the relationship between DNA methylation and exon expression are lacking. Thus, in this study, we compared the whole-genome patterns of DNA methylation at the exon level across two different tissues in *C*. *gigas*. In addition, we assessed the relationship between exon methylation and the corresponding exon mRNA expression in these tissues. The results of this study might enhance our understanding of the function of DNA methylation at the exon level in mollusks.

## Results and discussions

### Gene-level DNA methylation in *C*. *gigas*

To investigate the level of DNA methylation in *C*. *gigas*, and the association between methylation and exonic expression, we retrieved single-base resolution DNA methylation data from two different tissues: male gametes [32] and mantle [33]. Only BS-seq data of mantle tissue from the wild individual reported by Wang et al. could be used, because RNA-seq data were only available from the same tissue in that experiment for this individual [29]. In the present study, we remapped bisulfite-treated DNA sequence reads of these two tissues to the oyster genome using the software Bismark [35]. After read mapping and subsequent filtering, the average depth was approximately 17× (mantle) and 14× (male gametes; S1 Table). About 55.9 and 50.2% of CpG dinucleotides were covered by at least five unique reads in the mantle and male gametes (Table 1). In order to obtain the precise methylation state, only cytosines in CpG dinucleotides with a sequencing depth of more than five reads were retained for further analysis.

**Table 1:** Summary of coverage and mCpGs in the three tissues.

Sample	No. of CpG sites with coverage ≥5	Ratio (%)	No. methylated CpG s	Ratio (%)
Mantle	11,155,437	55.9%	2,186,754	11.0%
Male gametes	10,009,044	50.2%	2,044,344	10.2%

We identified 2,186,754 (mantle) and 2,044,344 (male gametes) methylated CpGs for the two tissues, respectively (Table 1). The number of genes with more than one methylated CpG was 18,376 and 17,229 for the mantle and male gametes, respectively. We identified 20,069 methylated genes (with more than one methylated CpG) in at least one tissue, 77.4% (15,536 genes) of which were methylated in both tissues. We found a higher fraction of genes with methylation level lower than 0.1 for the two tissues (S1 and S2 Figs). About 41.7% (mantle) and 42.7% (male gametes) of genes had methylation level lower than 0.1. In the following analysis, in order to avoid the deviation of genes due to low methylation level, we only used the genes having methylation level higher than 0.1 in at least one tissue.

### The relationship between DNA methylation and gene expression

**Fig 1:**
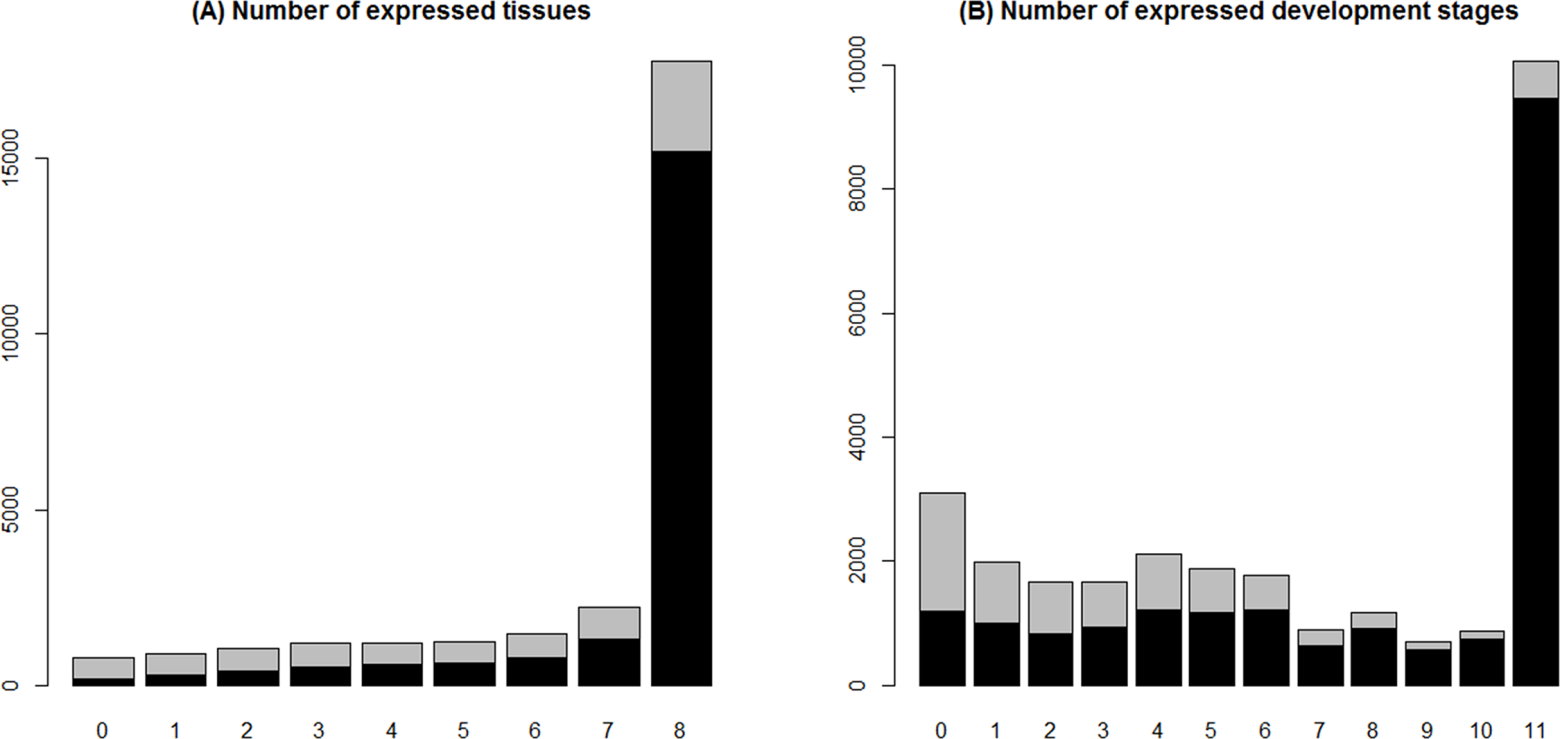
The distribution of DNA methylation and gene expression breadth. **A.** Stacked barplot for expressed methylated and non-methylated genes in different tissues. Black: methylated genes; Gray: unmethylated genes. B. Stacked barplot for expressed methylated and non-methylated genes at different development stages.

To determine whether methylated genes are expressed in more tissues and development stages than unmethylated genes, we first grouped the methylated genes in the two different tissues and termed this the methylated gene set. Then, we found that approximately 59.5% of the methylated genes were expressed (with an expression value larger than one) in all eight tissues, whereas only 16.3% of unmethylated genes were expressed in all tissues (Fig 1A). We obtained similar results for the 11 developmental stages, whereby, approximately 47.4% of the methylated genes were expressed in all developmental stages, and only 7.4% of the unmethylated genes were expressed in all stages (Fig 1B). Taken together, our findings are consistent with previous analyses showing that methylated genes are expressed at higher levels and in more tissues and development stages in *N*. *vitripennis* [19]. We confirmed these observations in *C*. *gigas*, indicating the conserved roles of gene body methylation on gene expression regulation and in the determination of the gene expression breadth and temporal variation.

### Exon length is associated with exon methylation status

**Fig 2:**
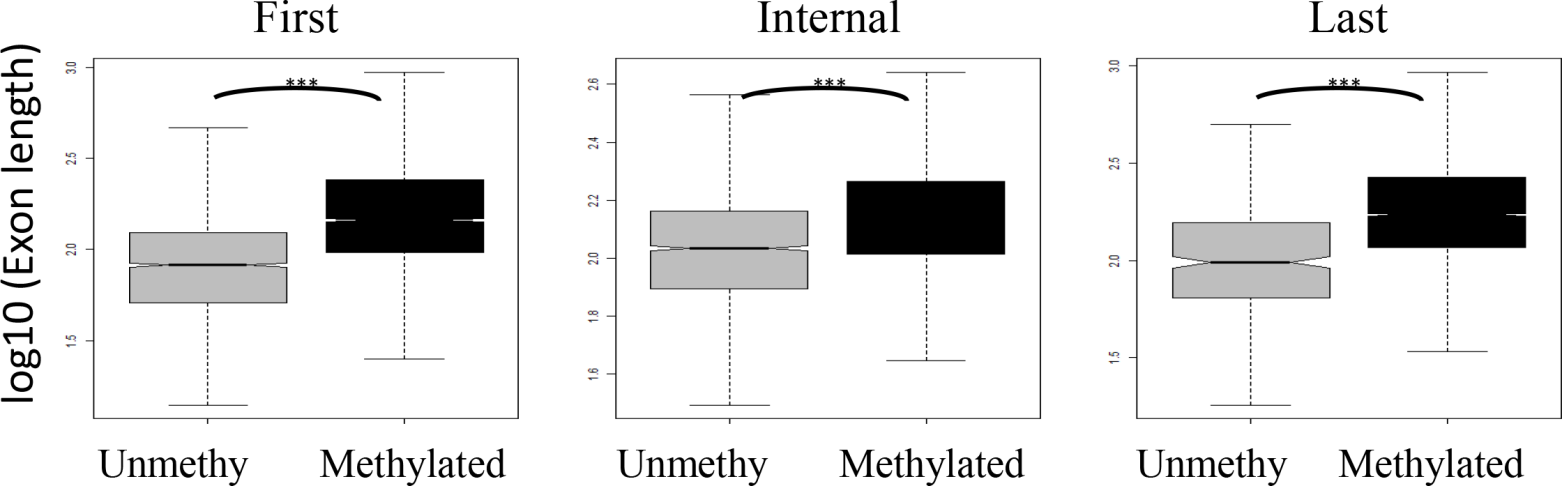
Exon methylation status is associated with exon length. Boxplot showing the exon length for methylated and unmethylated exons in male gametes. The plot illustrate that the methylated exons are longer than the unmethylated exons for all three types of exons. (***Wilcoxon rank-sum test *P* < 0.0001).

It has been reported that genes with high methylation levels are significantly longer than that those with low methylation levels in oyster [33]. Next, we investigated whether a positive relationship exists between DNA methylation and exon length at the level of exons, rather than whole genes. We focused on the exons of methylated genes, and then divided the exons into methylated (i.e. exons targeted by at least one identified mCG [methylated CpG sites]) and unmethylated groups. Because the first, internal, and last exons have different length distributions, we considered them separately and observed that methylated exons were significantly longer than unmethylated exons (Fig 2 and S3 Fig, Wilcoxon rank-sum test, *P* < 0.001). As exons represent the functional units of genes, our observation show that genes with high methylation levels are longer from the view of the exon levels in oyster.

### Distribution of DNA methylation in exons

**Fig 3:**
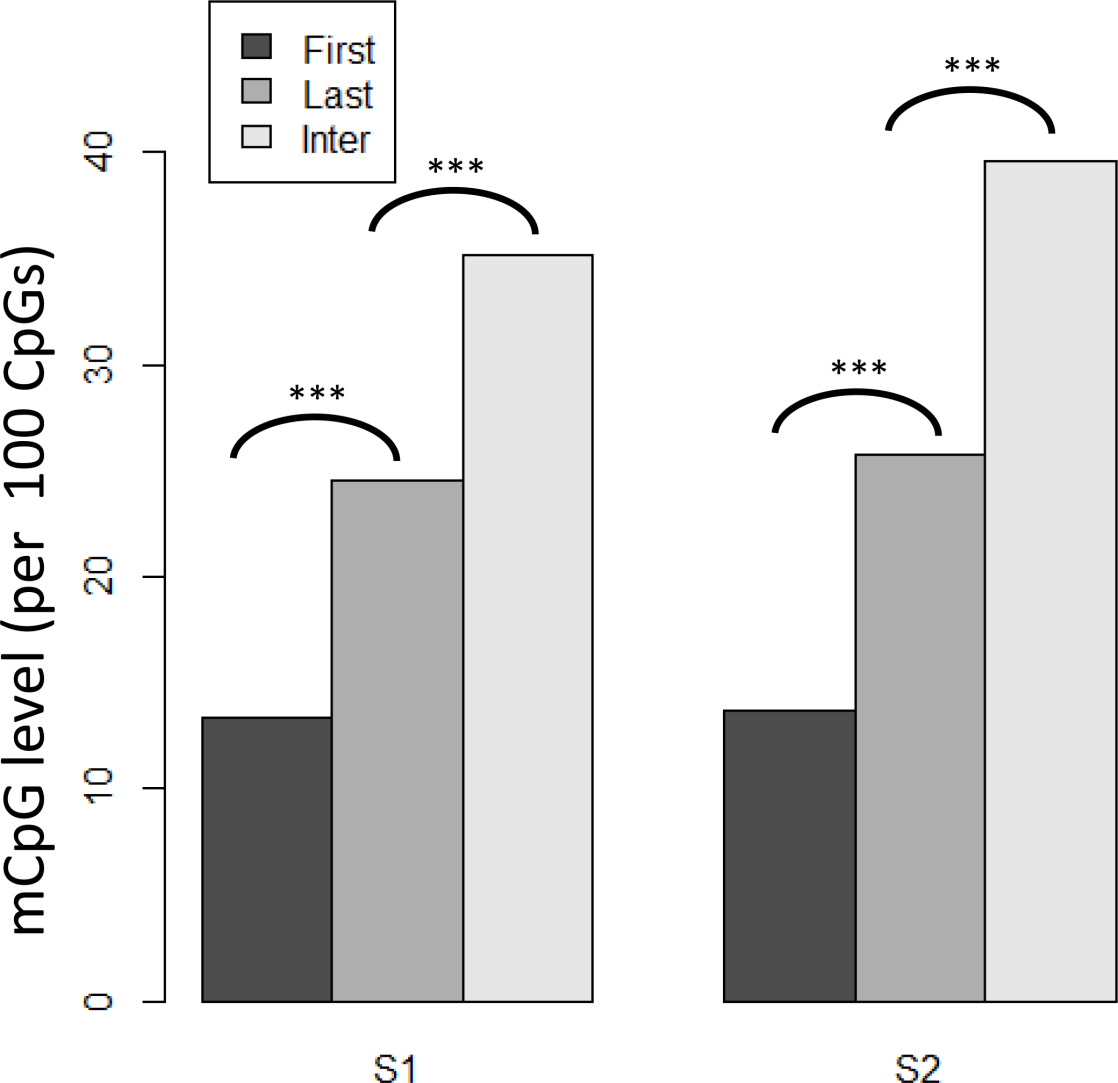
Methylation levels of the three types of exons. The methylation levels of three different types of exons (first, internal, and last) in the two tissues. (***Wilcoxon rank-sum test *P* < 0.0001)

DNA methylation is unevenly distributed in different genic regions in *C*. *gigas* (which was not determined for individual exons) [33]. In this study, we divided coding exons into three groups; first, internal, and last exons, and compared the methylation levels among these three groups. We show that in the two tissues studied, the lowest methylation level occurs in the first exons, followed by the last exons, and finally the internal exons (Fig 3). This result was consistent with previous findings in humans [36], and with the observation that methylation declines near the transcriptional start sites in *C*. *gigas* [33]. In contrast to the observation that the methylation level remained at a plateau along the gene bodies in *C*. *gigas* [33], in our study, we found that internal exons had a significantly higher methylation level than the last exons (Wilcoxon rank-sum test, *P* < 0.001). Our results provide a finer evaluation of the methylation pattern among exons in different relative positions in *C*. *gigas*.

### The relationship between DNA methylation and exon expression

**Fig 4:**
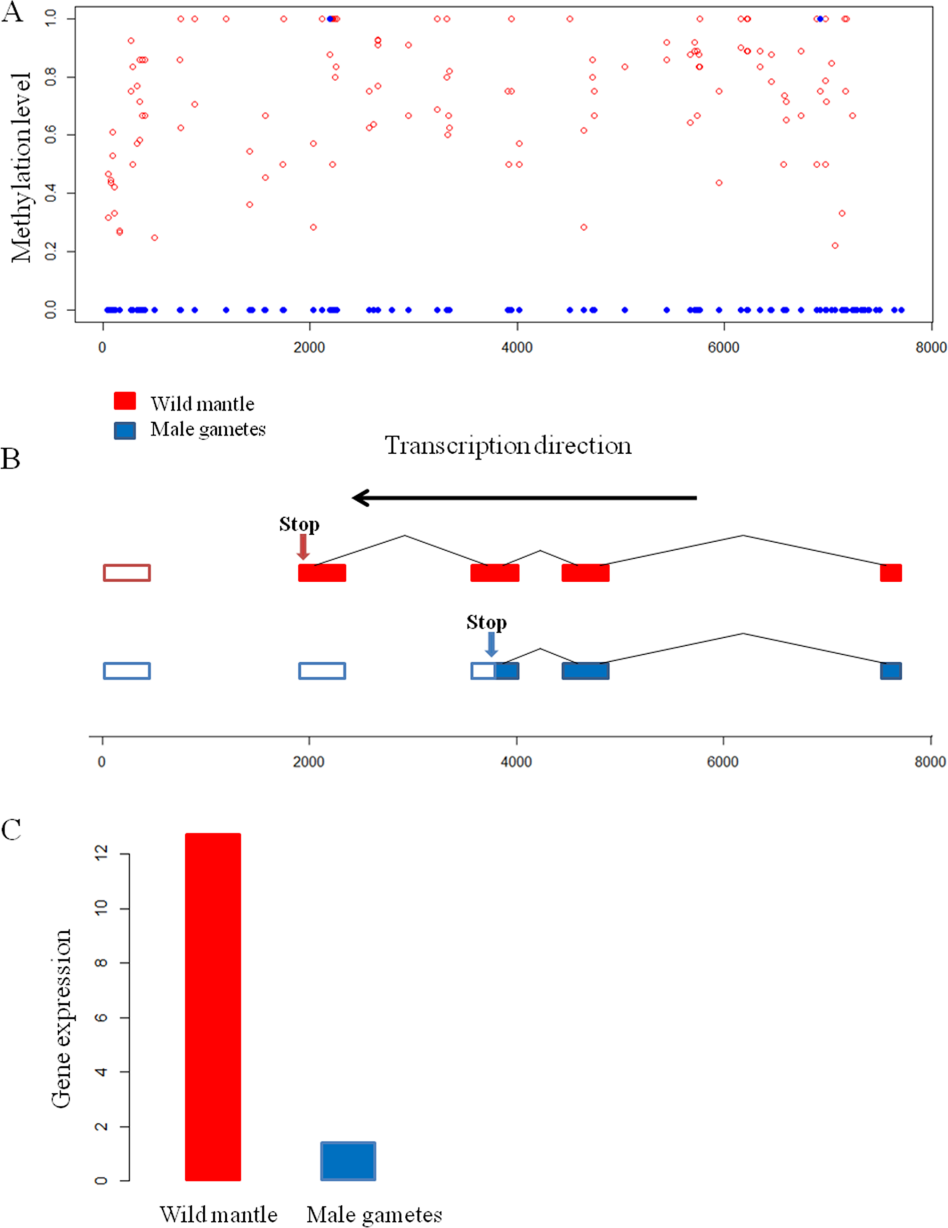
Expression profile of an alternatively spliced and differentially methylated gene CGI_10021620 in mantle and male gametes. (A) The CpG methylation pattern showing the level of methylation for individual CpGs in the two tissues. (B) Gene model of CGI_10021620 showing the two spliced variants for the two tissues. (C) Gene expression in the two tissues.

Given the suggested association between exon expression and DNA methylation in honeybees [34] and humans [36], we next tested whether a positive relationship exists between DNA methylation and expression at the exon level in *C*. *gigas*. To address this, we analyzed BS-seq and RNA-seq data among the first, internal, and last exons within genes that are both methylated (methylation level ≥0.1) and expressed (FPKM ≥1). We calculated the distribution of DNA methylation across the start and end sites for the three different groups of exons that were either included or skipped during transcription. We found that the exons included in the gene transcript contained significantly higher levels of DNA methylation than skipped exons from the exon start sites to end sites (Fig 4).

To explore the relationship between alternative splicing and expression patterns, we examined the gene CGI_10021620 in greater detail, which has different methylation levels and gene expression levels in male gametes and mantle. Fig 4 shows the distribution of mCpGs against the CGI_10021620 gene model (Fig 4A and 4B) and the relative expression of two spliced variants in both tissues (Fig 4C). This gene encodes a long protein in mantle tissue, and a short protein in male gamete tissue. The suggested DNA methylation pattern may function in the outcome of alternative splicing of this gene in *C*. *gigas*.

**Fig 5:**
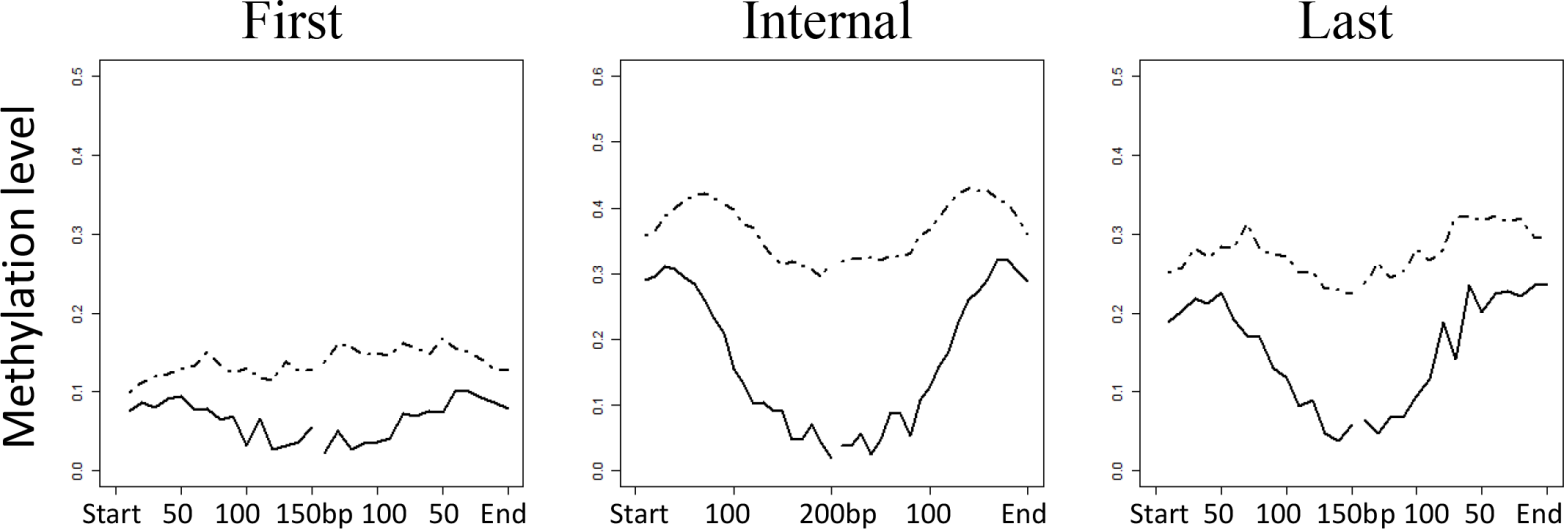
The distribution of DNA methylation among included versus skipped exons in male gametes. The plots illustrate that exons included in transcription are more highly methylated than those skipped during transcription for the first, internal, and last exons. The methylation level (weighted methylation level) around exons was calculated by dividing the regions +/− 200 bp within the start and end site of either included exons or skipped exons into 20 equal intervals for internal exons. For first and last exons, regions of +/− 150 bp were divided into 15 equal intervals. Solid line: included exons; dotted line: skipped exons.

In oyster, the DNA methylation level reportedly increases sharply from the transcription start sites, remaining at a plateau across the gene body until the transcription termination sites [33]. However, the pattern of DNA methylation across exons has not been determined. In this study, we observed that the levels of DNA methylation increased slowly from the start and end sites of the exons and then decreased rapidly towards the middle of exons (Fig 5 and S4 Fig). These patterns were significant for the internal and last exons.

Our observations in oyster are consistent with those in honeybee, whereby exons had higher methylation levels than skipped exons [34]. Furthermore, we provide further information on the patterns of DNA methylation across exons in oyster. We observed that the start and end sites of exons had higher levels of methylation than the middle sites, suggesting that DNA methylation might affect alternative splicing. Although the mechanism through which DNA methylation affects alternative splicing is unclear, this positive association led us to investigate a possible relationship between DNA methylation and the formation of alternatively spliced genes.

## Conclusion

In this study, we provide a comprehensive description of DNA methylation at both the gene and exon level in *C*. *gigas*. First, we found that methylated genes are expressed in more tissues and development stages than unmethylated genes, which has also observed in *N*. *vitripennis*. Then, we investigated the relationship between exon methylation status, exon length, and exon expression. We found that methylated exons are significantly longer than unmethylated exons, for first, internal, and last exons respectively. We also found that the exons included in the gene transcript contained significantly higher levels of DNA methylation than skipped exons, and the start and end sites of exons had higher methylation levels than the middle sites, suggesting that DNA methylation might affect alternative splicing.

## Materials and methods

### BS-seq data

We used BS-seq data sets of *C*. *gigas* from two tissues (mantle and male gametes) generated by Wang et al. (2014) and Olson et al. (2014). These data sets are deposited in the NCBI GEO database (accession number, GSE40302) and NCBI Sequence Read Archive (accession number, SRX390346). The oyster gene annotations and the corresponding coding sequences were downloaded from the NCBI (http://www.ncbi.nlm.nih.gov/). According to the relative positions of exons in the annotated genes, the retrieved coding exon regions were divided into three groups: first, internal, and last exons. Genes with a single exon or two exons were excluded.

### BS-seq analysis

In our study, we remapped the BS-seq reads to the reference genome (GenBank accession No., GCA_000297895.1) by using Bismark [35]. The bismark_genome_preparation command was used to prepare the reference genome, followed by the use of the bismark command to map BS-seq reads to the reference genome (parameters: “—multicore 12—bowtie2—temp_dir./temp/”). The bismark_methylation_extractor command was used to extract methylation information from the mapped results (parameters: -p—bedGraph—counts). The coverage2cytosine command was used to report the methylation results for each cytosine site with the parameter settings “-CX”.

### CpG methylation quantification

Methylated CpGs (mCpGs) are defined as CpG sites with ≥ 10% methylated Cs and ≥ 5 coverage. To accurately quantify the methylation percentages, we only included CpGs sites with a coverage of ≥5 (covered CpGs). This definition requires at least two unconverted C-containing reads to call a site methylated; therefore, a single T to C Illumina sequence error will not result in a spurious methylated site [19].

We used the metric, weighted methylation level, in covered CpGs to quantify the CpG methylation level in a particular region [37]. The weighted methylation level is defined as $\sum_{i=1}^{n}Ci/\sum_{i=1}^{n}\left(Ci+Ti\right)$, where C = reads supporting methylated cytosine, T = reads supporting unmethylated cytosine, i = position of cytosine, n = total number of cytosine positions. In the calculation, C was used to represent zero when the cytosine was unmethylated.

### Transcriptome data analysis

The transcriptome data sets used to compare gene expression in the two tissues were from the same tissues and same experiment as used in the BS-seq data analysis (accession numbers, GSE31012 and SRX390346). HISAT2 v2.0.4 package [38] was used to map RNA-seq reads to the oyster genome (GenBank accession No., GCA_000297895.1) using default settings. The SAM files produced by HISAT2 were converted to BAM files and sorted by SAMtools v.1.3 [39].

StringTie v1.3.3 package [40] processes the read alignments and the reference annotation with parameters setting “-eB”. Using this input, StringTie estimates abundances and creates new transcript tables for input to Ballgown. Gene expression levels were measured using FPKM calculated using R package Ballgown [41].

### Measurement of exonic expression level

The exonic expression levels were determined by counting the fragments per kilobase of exon per million mapped reads (FPKM) value for each exonic region in each examined sample. Of note, only the reads mapped to a unique location in the genome were selected to quantify exonic expression levels.

## Supporting information

S1 Fig DNA methylation level for each gene in mantle tissue. (TIF)

S2 Fig DNA methylation level for each gene in male gamete tissue. (TIF)

S3 Fig Exon methylation status is associated with exon length in mantle tissue. (TIF)

S4 Fig The distribution of DNA methylation level among included exons vs. skipped exons in the mantle tissue. (TIF)

S5 Fig The bioinformatics analysis pipelines for the WBS-seq and RNA-seq data. (TIF)

S1 Table Statistics of the BS-seq for each tissue. (XLSX)
