# Supplementary figures and images for: The association between DNA methylation and exon expression in the Pacific oyster Crassostrea gigas
Source: PLoS One. 2017 Sep 25;12(9):e0185224. doi: 10.1371/journal.pone.0185224 (PMC5612690; doi:10.1371/journal.pone.0185224)

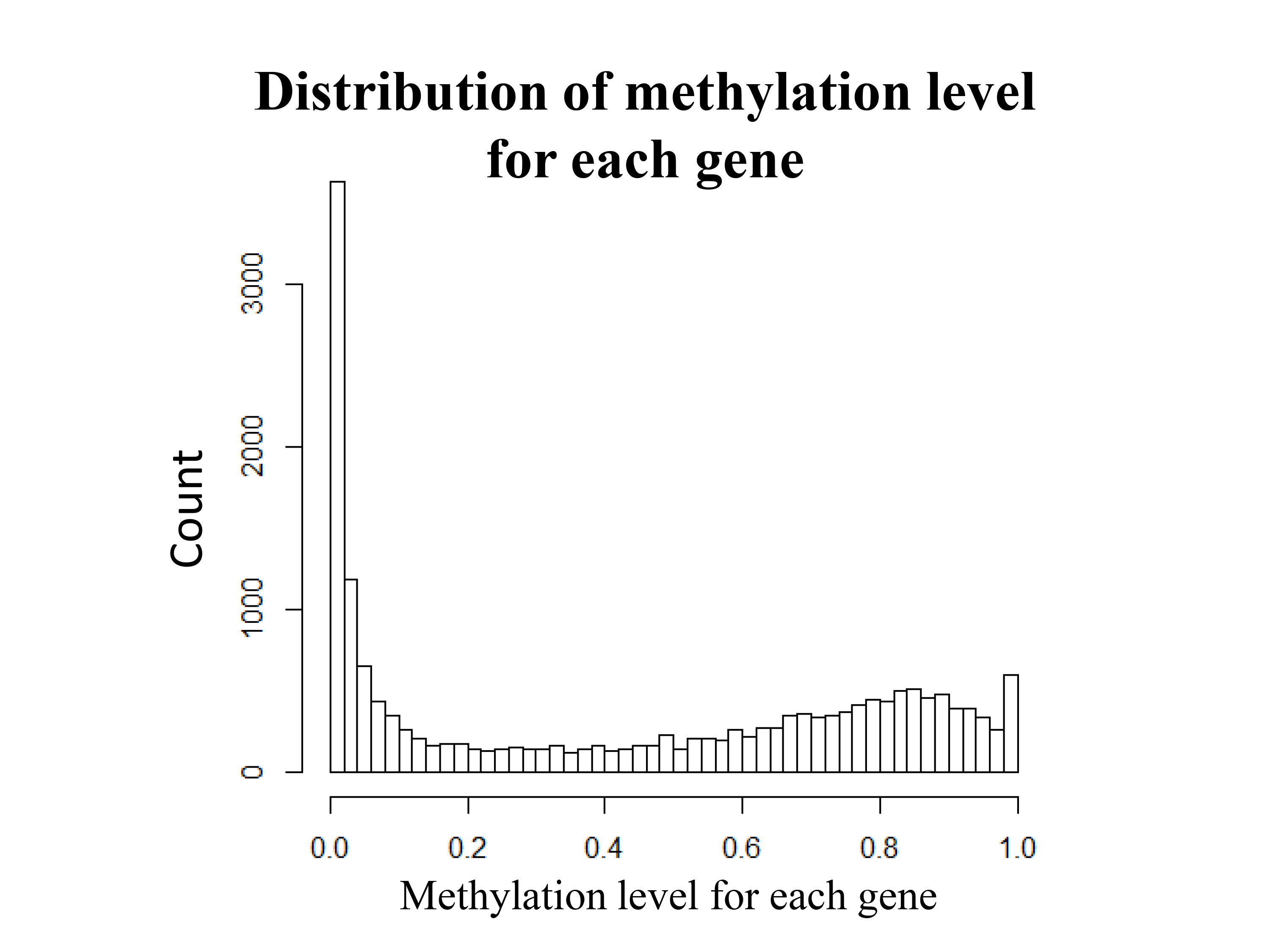

Supplement: S1 Fig — (TIF) [file pone.0185224.s001.tif]

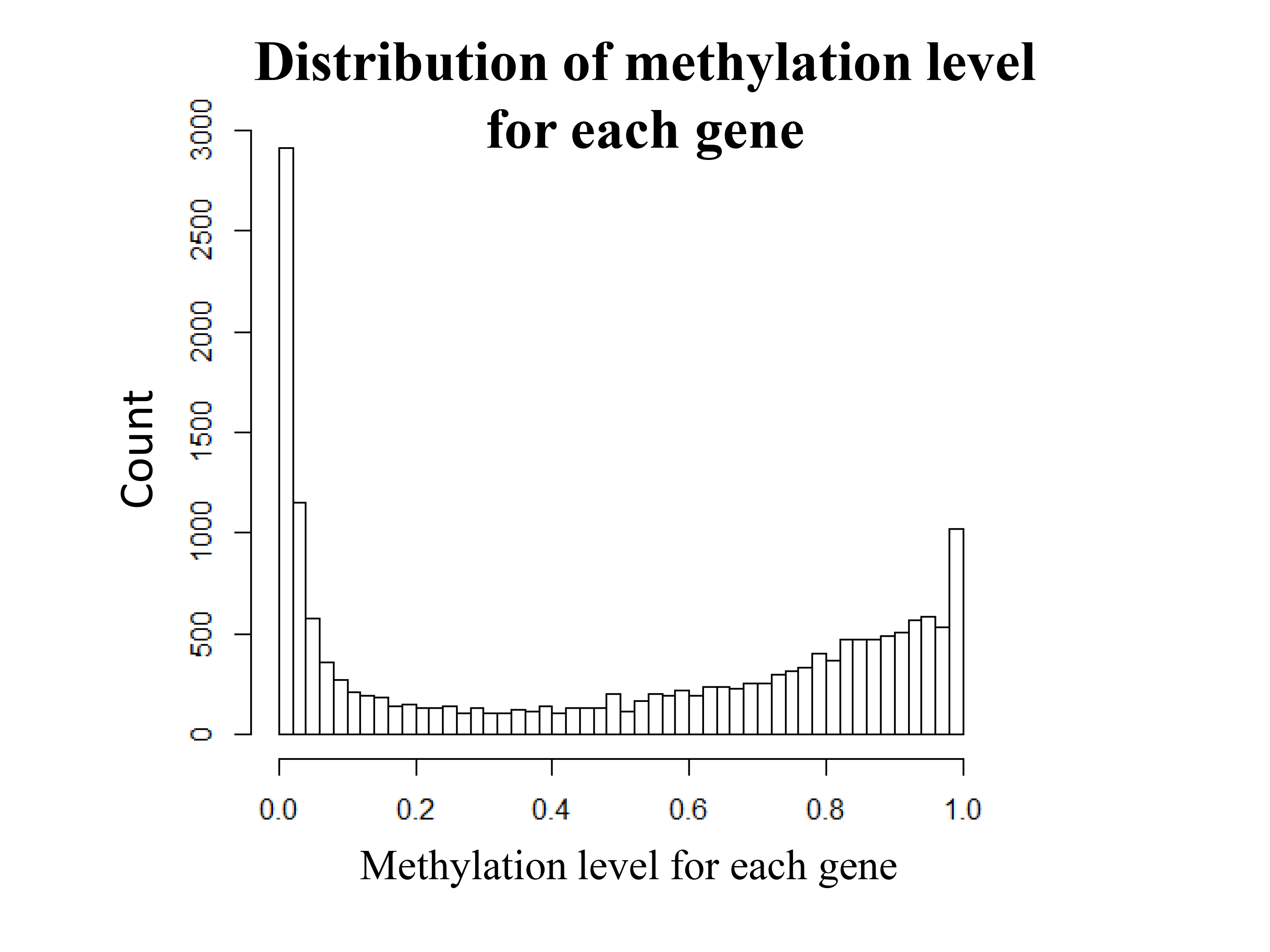

Supplement: S2 Fig — (TIF) [file pone.0185224.s002.tif]

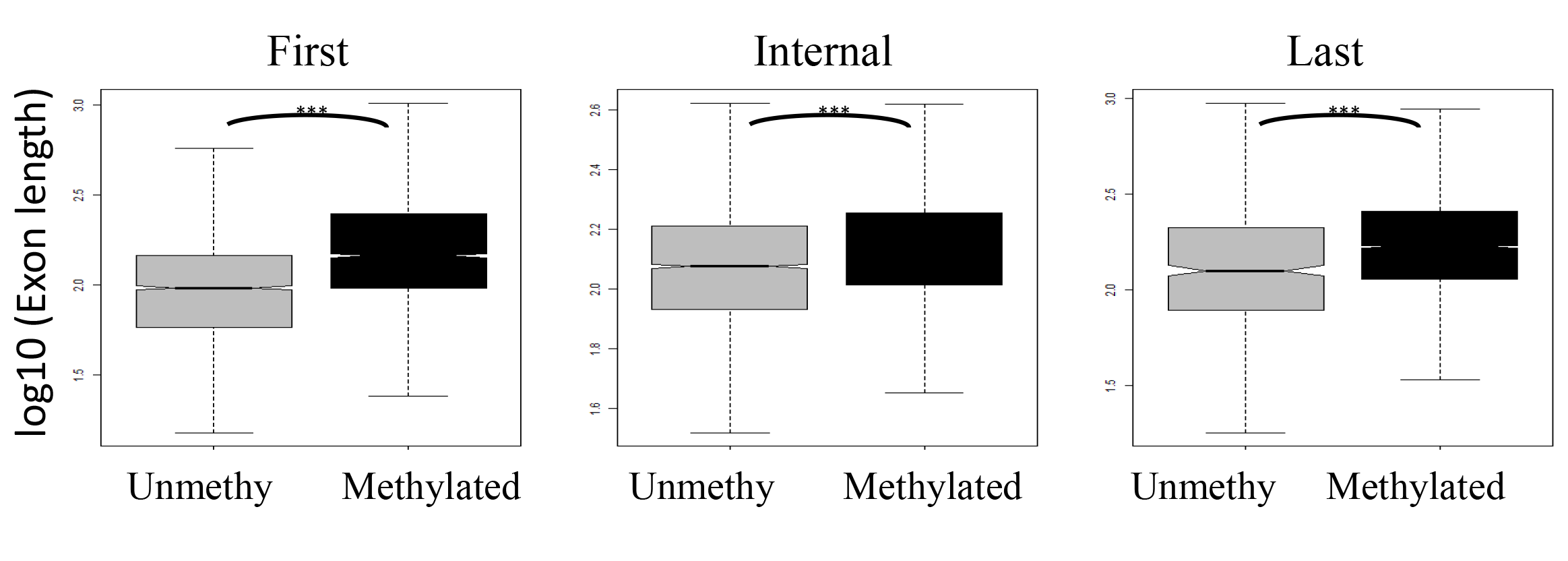

Supplement: S3 Fig — (TIF) [file pone.0185224.s003.tif]

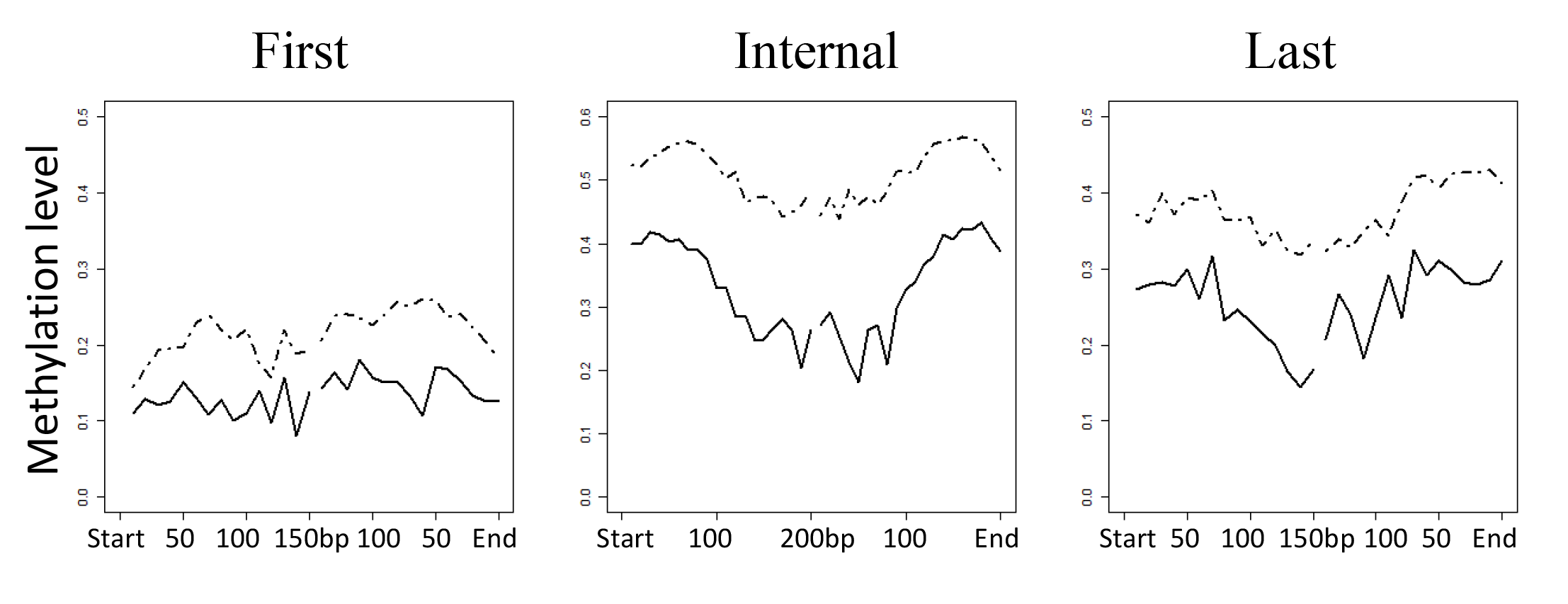

Supplement: S4 Fig — (TIF) [file pone.0185224.s004.tif]

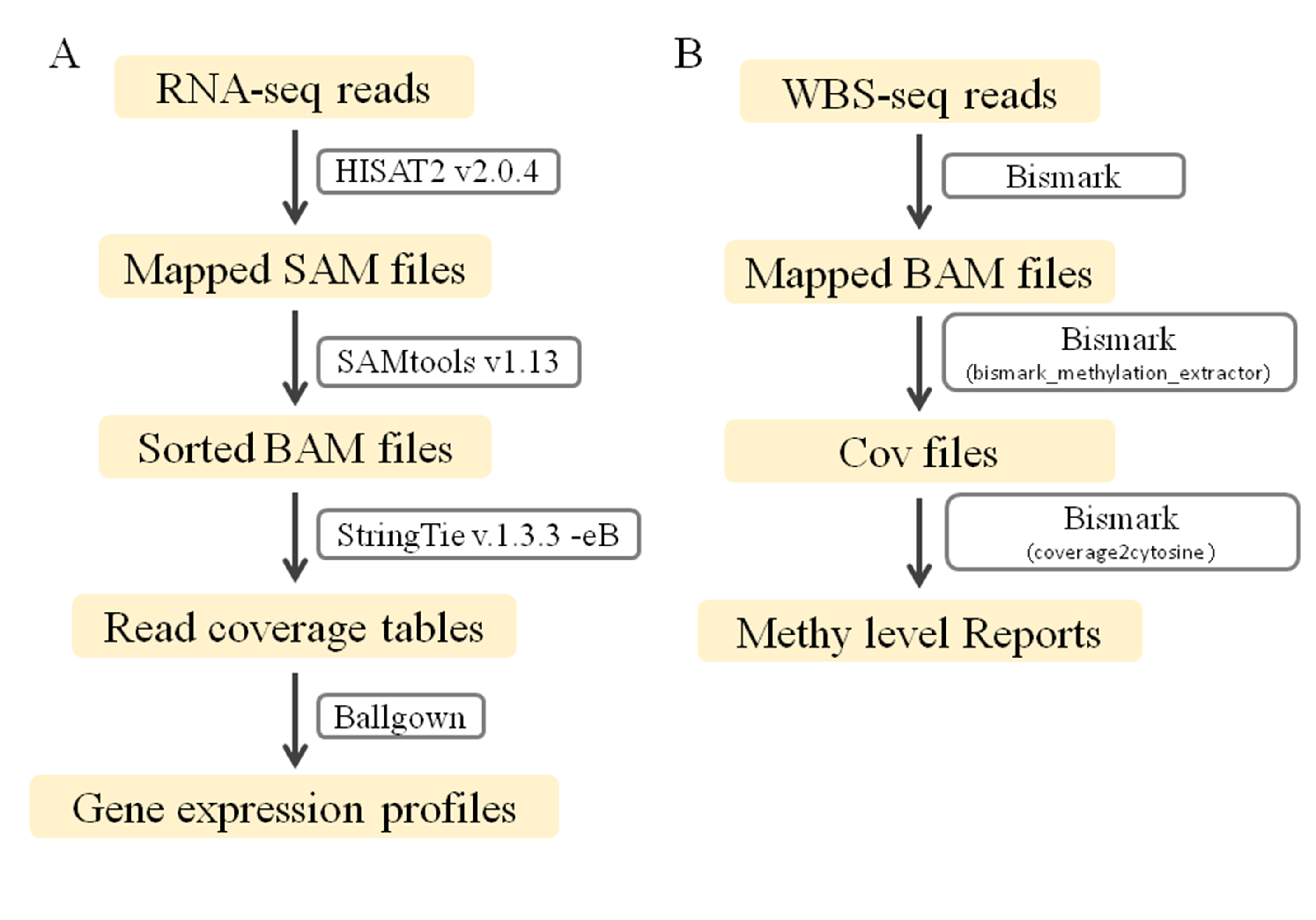

Supplement: S5 Fig — (TIF) [file pone.0185224.s005.tif]
